# A primer on prime: A prime editing update from advances to first-in-human trial

**DOI:** 10.1016/j.ymthe.2026.04.033

**Published:** 2026-04-20

**Authors:** Caleb Lushington, Paul Thomas, Fatwa Adikusuma

**Affiliations:** 1 School of Pharmacy and Biomedical Science, College of Health, Adelaide University, Adelaide, SA 5000, Australia; 2 South Australian Health and Medical Research Institute (SAHMRI), Adelaide, SA 5000, Australia; 3 South Australian Genome Editing (SAGE) Facility, SAHMRI, Adelaide, SA 5000, Australia; 4 Robinson Research Institute, Adelaide, SA 5000, Australia

**Keywords:** prime editing, CRISPR, genetic disease, genome editing, pegRNA, PE-integrase, off-target profiling, prime editing nuclease, dual-pegRNA

## Abstract

The advent of CRISPR systems has transformed genome editing, offering unparalleled efficiency and versatility with wide therapeutic potential. However, conventional CRISPR systems face key limitations, including unpredictable and imprecise outcomes during repair of double-stranded breaks and reliance on specific protospacer adjacent motif sequences. In response, prime editing (PE) has emerged as a powerful alternative, enabling precise custom edits using a fusion of Cas9 nickase and an engineered reverse transcriptase (RT) together with a PE guide RNA (pegRNA) that encodes the desired repair template. PE enables edits to be installed at or downstream of the target site, expanding the range of targetable sequences. Since its inception, PE has undergone extensive optimization, including Cas variant selection, RT engineering, and pegRNA improvements. In parallel, advances in delivery, including nanoparticles and split viral systems, have accelerated translation across preclinical disease models. Notably, PE has now entered the clinic, with the first-in-human study reporting functional restoration with a promising safety profile to date. Here, we summarize recent mechanistic insights, architectural innovations, and therapeutic applications of PE and discuss the remaining challenges in efficiency, delivery, and safety that will shape broader clinical impact.

## Graphical abstract

**Figure:**
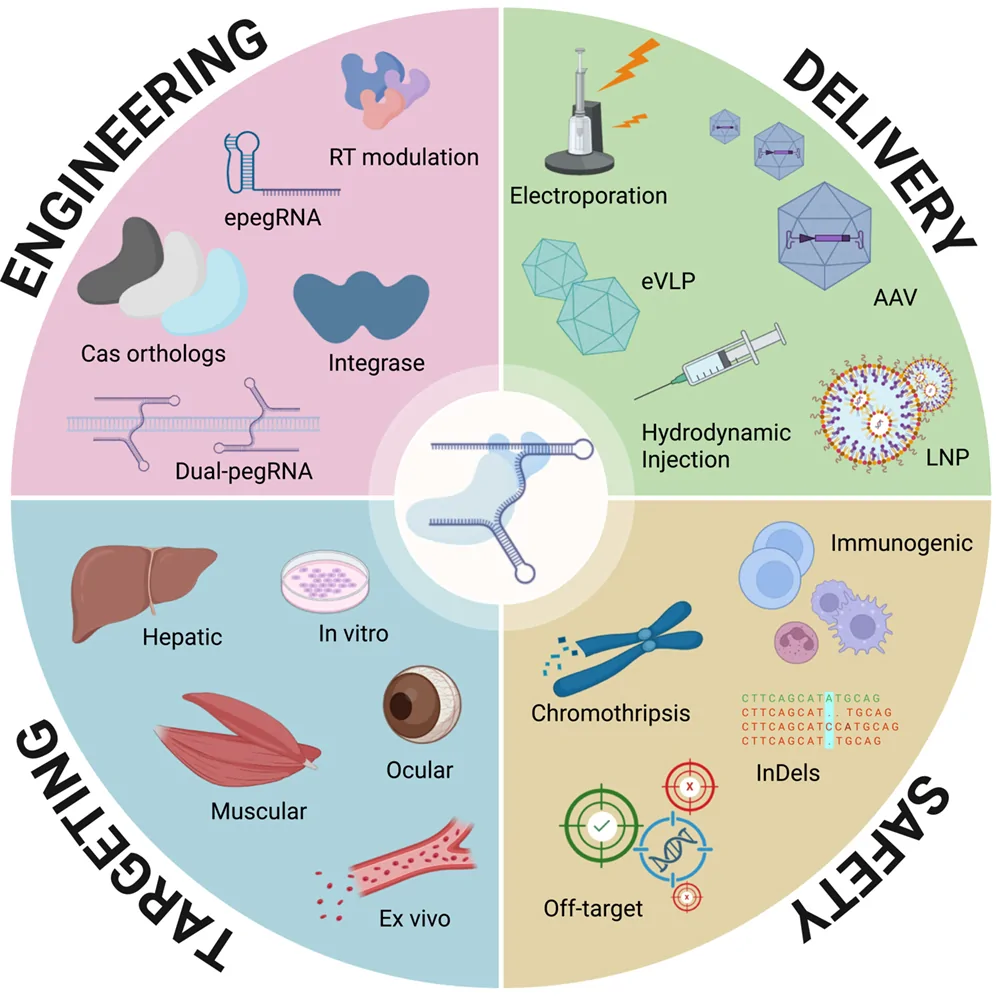

## Teaser

Lushington and colleagues synthesize the rapidly evolving prime editing landscape, from pegRNA and editor engineering to dual-pegRNA and integrase/recombinase platforms, and connect these advances to safety frameworks and the first-in-human study. The review provides a clinically oriented roadmap for translating precision genome rewriting into therapies.

## Introduction

The ability to perform targeted genome manipulation has transformed the biomedical field, offering the power to rewrite DNA with unprecedented ease. At the heart of this revolution is the ability to interrogate and correct pathogenic mutations that drive genetic disease, from single-base substitutions to large structural variants. While early genome editing tools such as zinc finger nucleases (ZFNs) and transcription activator-like effector nucleases (TALENs) demonstrated the feasibility of targeted DNA manipulation, their labor-intensive protein engineering and limited flexibility restricted therapeutic potential.^1^

The emergence of CRISPR-Cas systems revolutionized genome editing by providing a simple, programmable nuclease platform. The most widely used system, CRISPR-Cas9, requires two targeting components: a single guide RNA (sgRNA) of ∼20 nucleotides (nt) that directs the nuclease to complementary genomic DNA and recognition of a short protospacer adjacent motif (PAM) sequence by Cas9. Cas9 contains two nuclease domains that introduce a double-stranded break (DSB) at the target site once PAM recognition and gRNA binding occur.^2^^,^^3^ However, the outcome of DNA repair depends entirely on endogenous pathways, most commonly error-prone non-homologous end joining (NHEJ) or microhomology-mediated end joining, which introduce small insertions or deletions (indels). While these indels can be useful for gene disruption, therapeutic correction of pathogenic mutations requires more precise editing. This can be achieved through homology-directed repair (HDR) when a donor template with homology arms (HAs) is supplied. However, HDR is inefficient in most mammalian cells and is largely restricted to S and G2 phases of the cell cycle, limiting its therapeutic utility.^4^^,^^5^ To bypass reliance on HDR, base editors were developed by fusing catalytically impaired Cas proteins to deaminase enzymes. Base editors mediate efficient, programmable nucleotide conversions without creating DSBs. Cytosine base editors (CBEs) convert C→T and adenine base editors (ABEs) convert A→G within a defined editing window. These systems substantially broadened the scope of CRISPR editing but are limited to transition mutations, leaving many pathogenic variants—particularly those requiring precise insertions, deletions, or transversions—inaccessible.^6^^,^^7^ Moreover, because all compatible nucleotides within the editing window are modified, base editors often lack precision and introduce bystander edits that can limit therapeutic utility.

**Figure 1:**
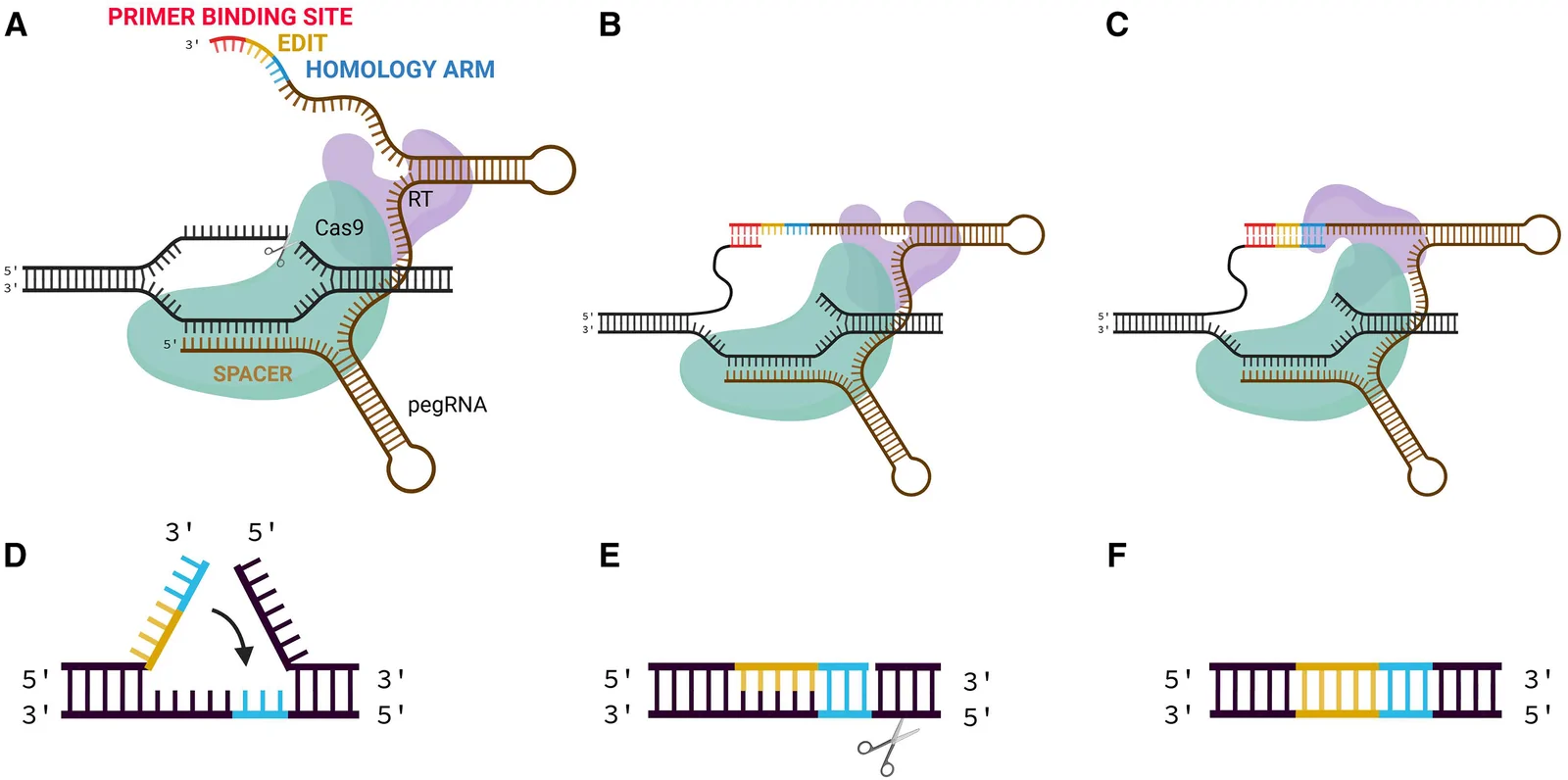
Prime editing components and mechanism PE, consisting of a Cas9 (green) and reverse transcriptase (purple), located at a target DNA site (black) by the pegRNA (brown), including the primer binding site (red) and reverse transcriptase template, consisting of the intended edit (yellow) and homology arm (blue) (A). Once the spacer has annealed the target site and a nick is introduced, the PBS can associate upstream of the cut site (B) and prime the reverse transcriptase to transcribe the repair template into the break (C). Competition between the newly synthesized flap and the WT flap for binding the target site ensues (D) until the edited flap is integrated and the WT flap is excised. A second nick introduced downstream of this (E) can improve the edit integration across both DNA strands (F). Created with BioRender.com.

Seeking to overcome these limitations, Andrew Anzalone and David Liu developed prime editing (PE), a next-generation CRISPR-based genome editing system.^8^ PE tethers a DNA-nicking H840A Cas9 nickase (nCas9) to an engineered Moloney Murine Leukemia Virus (M-MLV) reverse transcriptase (RT). The system uses a PE guide RNA (pegRNA), which contains both the conventional spacer sequence for Cas9 targeting and a 3′ extension comprising a primer binding site (PBS) and an RT template (RTT) encoding the desired edit and HA (Figure 1A). Due to the flexibility of RTT length, edits can be introduced at or proximal to the cut site, broadening the targetable range of sequence beyond the traditional CRISPR cut site.

Upon PAM recognition and target binding, nCas9 nicks the non-target strand, exposing a 3′ end that hybridizes to the PBS (Figure 1B). This primes reverse transcription, generating a 3′ DNA flap containing the programmed edit and HA (Figure 1C). The edited flap competes with the original 5′ flap to anneal to the complementary strand; successful incorporation requires the edited flap to replace the wild-type (WT) sequence, after which the complementary strand is resolved to incorporate the edit across both strands (Figure 1D). This form of PE is referred to as PE2.

PE efficiency can be further improved by introducing a nick on the complementary strand 40–90 bp downstream of the intended edit using an sgRNA (Figure 1E). This promotes repair using the edited strand as a template, thereby increasing the likelihood of edit incorporation across both DNA strands (Figure 1F)—a strategy known as PE3. A variation, PE3b, positions the nicking sgRNA such that it recognizes only the edited allele, reducing unintended DSB formation caused by simultaneous nicking of both strands.^8^ While PE3 improves efficiency, it can also increase indel byproducts. In practice, PE efficiencies are robust for small edits in laboratory cell lines such as HEK293T, but efficiency is highly variable across different cell types and declines with larger sequence modifications.^8^^,^^9^^,^^10^^,^^11^ This variability highlights the importance of ongoing mechanistic studies and optimization to expand the range of clinically relevant applications.

## Optimizations to improve PE performance

Although PE offers broad versatility, early versions of the system were hampered by modest efficiency, variable fidelity, and a restricted editing window. Recent advances have therefore focused on optimizing PE performance at multiple levels—including pegRNA engineering, modulation of DNA repair pathways, enhancement of nuclear delivery, and direct engineering of the PE protein itself. Collectively, these strategies aim to improve efficiency, refine product purity, and expand the scope of targetable sites.

### RTT length and tertiary structure can dictate edit efficiency and precision

The design of the pegRNA is central to PE performance, as both the PBS and RTT strongly influence editing outcomes. High-throughput studies suggest that PBS and RTT lengths of approximately 13 and 12 bp, respectively, often provide optimal performance, though the precise requirements remain context dependent and may require locus-specific tuning.^12^ A central limitation of pegRNAs is their susceptibility to 3′ end degradation, which can remove the PBS and RTT extension while leaving spacer and scaffold sequences intact. These truncated guides continue to direct Cas9 cleavage but fail to initiate reverse transcription, resulting in indels and reduced fidelity.^13^ To address exonuclease degradation, engineered pegRNAs (epegRNAs) were designed by incorporating structured RNA pseudoknots (evoPreQ1 motifs) at the 3′ end. epegRNA motifs enabled protection of pegRNA extensions from 3′ exonuclease degradation, resulting in 3- to 4-fold improvement in correct editing.^13^ The PE7 system, created by tethering La, an exonuclease protection factor, to PEmax, further exploits the ability to stabilize 3′ polyU tails to increase efficiency across multiple sites.^14^

Beyond PBS and RTT length, additional design features critically shape pegRNA performance, particularly interactions that alter annealing affinity or cause over-extension of reverse transcription. Hybridization between PBS and spacer sequences can create auto-inhibitory interactions, rendering pegRNA inactive; optimizing the melting temperature (Tm) to ∼37°C for pegRNAs and ∼42°C for epegRNAs mitigates this effect.^15^^,^^16^ Reverse transcription can also extend into the scaffold hairpin, introducing unwanted scaffold sequence or disrupting HA complementarity.^8^^,^^17^^,^^18^^,^^19^ This can be alleviated by chemical modifications such as 2′-O-methylation or abasic spacers between the RTT and scaffold, which terminate over-extension.^20^ However, these modifications necessitate synthetic pegRNA construction and delivery, adding complexity. Alternatively, recoding the scaffold sequence to avoid homology with the target reduces the incorporation of scaffold bases.^9^

pegRNA design has also been leveraged to broaden the spatial range of PE, particularly enabling edits upstream of the nick site. The EXPERT system combines extended pegRNAs (ext-pegRNAs) with an auxiliary nicking sgRNA, permitting both upstream and downstream edits—including insertions and replacements of up to ∼100 bp.^21^ Similarly, actRNA:t and related dual-RNA uPEn (ubiquitin variant-assisted Prime Editing Nuclease) strategies have demonstrated alternative configurations of guide and template RNAs to drive edits into the target strand to permit upstream editing.^22^ CiPEs (Circularized Prime Editors) employ circularized pegRNAs with nCas9-D10A and the Rep-X helicase to improve stability and flap resolution, achieving upstream edits at otherwise refractory sites.^23^ Collectively, these innovations highlight pegRNA engineering as an important factor in precision, flexibility, and expanded editing capacity in PE.

### Modulating DNA repair pathways can enhance edit outcomes

While pegRNA optimization shapes the initiation of PE, the efficiency and fidelity of final outcomes are dictated by host DNA repair pathways. Among these, mismatch repair (MMR) factors play a particularly decisive role, as they recognize and excise short mismatches in nicked DNA, including the 3′ flaps generated during PE. MMR-proficient cells thus exhibit reduced editing, but inhibition of MMR regulators MLH1 and PMS2 consistently rescues efficiency.^9^^,^^10^^,^^11^ Strategies to mitigate MMR thus include application of dominant-negative variants such as MLH1dn (standardized as PE4/PE5 when combined with PE2/PE3), short hairpin RNA (shRNA) knockdown, degradation tags, or knockout lines. Rational pegRNA design also provides a means of evading MMR, with edits > 13 bp or strategically positioned mismatches (e.g., bases 1/4, 2/5, and 3/6 from the cut) evading rejection.^24^^,^^25^

Beyond MMR, nucleotide availability represents another constraint, particularly in quiescent or primary cells where low dNTP pools limit RT activity. Inhibition of SAMHD1, a dNTP-depleting triphosphohydrolase, combined with exogenous nucleotide supplementation, restores efficiency under these conditions.^26^^,^^27^ Exonuclease activity also critically shapes repair outcomes: 5′ exonuclease activity reduces competition between the newly synthesized 3′ flap and the WT 5′ flap, as demonstrated in the 5′-3′ T5 exonuclease-associated Exo-PE system.^28^ Similar improvements have been observed in plant cells, where T5 exonuclease improved efficiency by 1.7- to 2.9-fold.^29^ Conversely, TREX1/2 exonucleases degrade 3′ flaps and antagonize PE editing.^25^ Engineered Cas9 variants that reposition the nick site can also minimize flap competition by weakening Cas9 association with the nicked 5′ end. This enables degradation of the WT flap to favor integration of the edited strand while minimizing indels.^30^

Additional innovations targeted chromatin accessibility. Epigenetic conditioning provides yet another layer of control, with CRISPRa-mediated activation at target loci enhancing editing in a context-dependent manner.^11^ Collectively, these findings demonstrate how manipulating DNA repair, flap dynamics, and chromatin state can substantially elevate PE efficiency.

### Overcoming nuclear delivery of the large PE protein improves PE initiation

Even with optimized pegRNA and DNA repair modulation, efficient PE editing requires reliable nuclear import of the large PE protein (∼240 kDa). Enhancing nuclear localization signals (NLSs) has therefore proven to be a valuable optimization strategy. Variants such as PEmax (featuring N-terminal, RT-linker, and dual C-terminal NLS tags), PE∗ (dual N- and C-terminal tags), and iPE (three monopartite and two bipartite NLSs) all outperform standard PE, with PEmax showing superior efficiency to PE2∗ in side-by-side comparisons.^9^^,^^28^^,^^31^

### Engineering the PE protein can improve activity while reducing size

Beyond NLS tuning, direct engineering of the PE protein has produced variants with both higher correct editing activity and reduced size. Yeast-based directed evolution of PE components has identified beneficial substitutions in both nCas9 and RT, producing PE_Y18 with ∼3.5-fold higher precise editing activity than PEmax.^32^ Phage-assisted continuous evolution (PACE) of compact retroviral RTs and Cas9 domains identified functional variants up to 810 bp smaller in size or more efficient than PEmax, PE6a, and PE6b, respectively.^18^ Parallel efforts truncating the RT RNaseH domain reduced PE size by ∼600 bp without compromising efficiency, a modification particularly advantageous for delivery using packaging-limited vectors such as adeno-associated virus (AAV).^33^^,^^34^^,^^35^^,^^36^ Despite these advances, the overall size of PE remains a significant barrier to efficient delivery—particularly *in vivo*—and continues to be a major focus of ongoing optimization.

### Broadening PE targetability by expanding PAM recognition

While the pegRNA RTT extends the editable range of PE beyond the immediate cut site, edits positioned further from the nick are less efficient at integration, and PAM requirements still apply. As such, expanding PAM recognition from the canonical SpCas9 NGG motif has dramatically increased the targetability of PE. Engineered Cas9 variants such as Cas9-VQR (NGA), Cas9-NG, and SpG-Cas9 (NGN) broaden PE coverage, while SpRY-Cas9 approaches a near PAM-less state, enabling near genome-wide targeting.^37^^,^^38^^,^^39^^,^^40^^,^^41^ Additionally, compact orthologs, including SaCas9 (NNGRRT), SaKKH (NNNRRT), and CjCas9 (NNNNAH/NNNNNHA), extend flexibility at the cost of reduced efficiency.^9^^,^^31^^,^^42^ Cas12a has also been adapted for PE, exploiting its T-rich PAM preference (TTTV). Unique to this system, pegRNAs can be circularized and split into separate crRNA and RTT modules. This architecture improves stability and supports multiplex editing across up to four loci. Among tested configurations, nickase Cas12a paired with split circular pegRNAs shows the highest efficiency.^43^ Collectively, PAM-relaxed Cas9 variants and Cas12a systems extend PE coverage to ∼95% of pathogenic variants, significantly expanding PE capabilities.^41^

Together, these refinements have enhanced the activity, precision, and target range of PE, rendering the system more robust and adaptable (Table 1). While incremental optimizations have steadily improved efficiency and fidelity, the field is now turning toward strategies that fundamentally reconfigure the PE architecture, broadening its capacity beyond single-nucleotide edits to encompass larger and more complex genome modifications.

**Table 1:** Prime editing optimization summary

Feature	Optimization	Reference
pegRNA	optimal length: 12 nt RTT, 13 nt PBSoptimal PBS Tm: 37°C–41°C	Kim et al.,^12^ Koeppel et al.,^25^ Ponnienselvan et al.,^15^ Qian et al.^16^
	epegRNA tertiary motifs protect 3′ ends from degradation	Nelson et al.^13^
	upstream PBS for inverse, spacer spanning edits	Xiong et al.,^21^ Chen et al.,^22^ Liang et al.,^23^ Li et al.^24^
PAM flexibility	VQR (NGA), NG (NG), and SpG (NGN)	Kweon et al.^41^
	SpRY (NNN [NRN preference]), SaCas9 (NNGRRT), SaKKH (NNNRRT), evoCjCas9 (NNNNAH and NNNNNHA)	Chen et al.,^9^ Liu et al.,^31^ Walton et al.,^37^ Liu et al.^42^
	Cas12a (TTTV)	Liang et al.^43^
DNA repair	inhibitors of MLH1 and PMS2 reduce MMR	Chen et al.,^9^ Ferreira da Silva et al.,^10^ Li et al.^11^
	RTT silent mutations reduce MMR	Chen et al.,^9^ Li et al.^24^
	edits > 13 bp avoid MMR	Koeppel et al.^25^
	T5 5′-3′ exonuclease removes 5′ WT flap competition	Truong et al.,^28^ Liang et al.^29^
	altered Cas nick position permits 5′ flap degradation	Chauhan et al.^30^
	La exonuclease stabilizes pegRNA polyU	Yan et al.^14^
	CRISPRa ahead of target sites increases PE-DNA accessibility	Li et al.^11^
Nuclear delivery	NLSs improve PE entry into nucleus for improved editing efficiency	Chen et al.,^9^ Truong et al.,^28^ Liu et al.^31^
Protein engineering	directed evolution of optimal PE mutants	Doman et al.,^18^ Weber et al.^32^
	RT RNaseH truncation reduces PE size while maintaining efficiency	Grünewald et al.,^33^ Gao et al.,^34^ Zheng et al.,^35^ Böck et al.^36^

## Advanced PE platforms for broader editing applications

PE has benefitted from gradual refinements, but recent innovations represent a more fundamental reconfiguration of the system’s architecture. Rather than simply tuning components, these approaches reimagine how PE is executed, enabling entirely new classes of genome edits. Nuclease-based PE (PEnuc), dual-pegRNA strategies, and PE-integrase (PEI) platforms expand the scope of the technology from modest single-nucleotide changes to large insertions, deletions, and even programmable chromosomal rearrangements, thereby broadening the reach of PE into applications previously considered inaccessible.

### PEnuc systems

**Figure 2:**
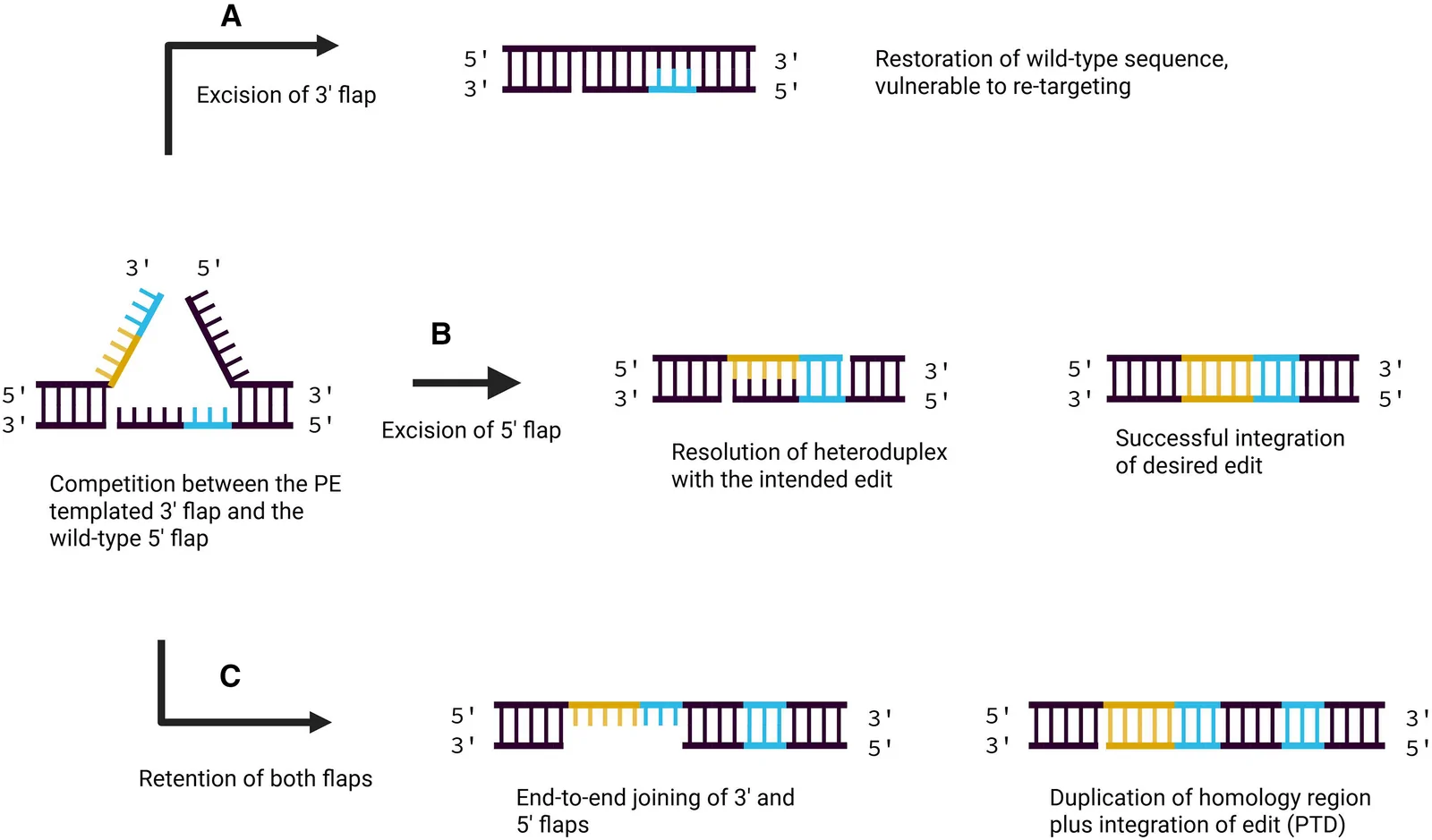
Repair mechanism of PE nuclease following dsDNA cleavage and RTT transcription into the cut site (A) One of three repair outcomes occurs. Competition between the 3′ and 5′ flaps can result in the displacement of the 3′ strand and restoration of the wild-type sequence; however, this is able to be re-targeted by PE nuclease. Alternatively, 5′ strand excision can prompt annealing between the 3′ strand HA and complementary target sequence (blue) for precise integration of the edit (B) or, if no excision of the 5′ flap or homology annealing occurs, an end-to-end joining event between the synthesized and wild-type flaps occurs for an imprecise integration of the edit (C). In the latter scenario, the presence of both an HA and a complementary target site results in a duplication of sequence, here termed a partial template duplication (PTD).

Initial PE systems relied on nickases to minimize the risks of DSBs, but this design limited performance in certain cellular contexts. Recent studies have therefore revisited WT SpCas9 nuclease within the PE framework, giving rise to PEnuc. While PEnuc increases the frequency of imprecise repair products, it also delivers markedly higher editing activity than PE nickase, including in settings where conventional PE fails, such as MMR-proficient or quiescent cells.^10^^,^^26^^,^^27^^,^^44^ The imprecise edits primarily consist of partial template duplications (PTDs), which are thought to result from the dominance of NHEJ, where blunt-end integration of the repair templated flap occurs without displacing the unedited target strand (Figures 2A–2C.). This highlights that while PEnuc transcribes edits efficiently, final resolution is strongly dependent on cell-specific repair preferences.

Our group demonstrated the practicality of the PEnuc system across multiple cell types, with 2- to 4-fold increases in activity observed in HeLa and K562 cells, largely comprising PTDs, and, in some cases, higher rates of correct editing in mouse ESCs compared to nickase PE.^44^ PEnuc has also proven particularly valuable in zygote editing, where nickase-based PE was previously inefficient. Here, cytoplasmic delivery of PEnuc mRNA enabled the efficient generation of multiple mutant mouse lines.

To mitigate the imprecision of PEnuc, co-delivery of DNA repair modulators has been explored. Factors that mediate the DNA repair pathway choice toward HDR likely increase (1) resection of the competing target flap and (2) annealing of the transcribed HA with the complementary target sequence, to resolve edits precisely (Figure 2B). Inhibition of DNA-PK, a central kinase that drives classical NHEJ, nearly eliminated PTDs in HEK293T cells, while inhibition of 53BP1, a DNA end-protection factor that blocks end resection and biases repair toward NHEJ, increased correct edits 4- to 8-fold across multiple human cell lines.^45^^,^^46^ Additional modulation of Polθ, an error-prone polymerase that promotes MMEJ, using small interfering RNA (siRNA) with M3814 (DNA-PK inhibitor) improved precision to levels comparable with PE3.^47^

Interestingly, alternative strategies have sought not to suppress NHEJ but to exploit it. Peterka et al.^45^ demonstrated blunt-end insertions without RTT-derived HAs that utilize a PBS and single-nucleotide template before RT termination at the scaffold. Leveraging the NHEJ mechanism achieved up to 50% efficiency for templated single-nucleotide insertions in HeLa cells.^45^ Although this bypassed PTDs, unintended scaffold-derived insertions were introduced. Peterka et al.^45^ addressed concerns about off-target editing with PEnuc, which was found to be higher than standard PE but equivalent to traditional Cas9 approaches. However, it was also discovered that combining small insertions with templated deletions can produce fewer large deletions than the Cas9 nuclease.^45^^,^^48^ Moreover, by avoiding the MMR surveillance that limits PE nickase, PEnuc expands editing applicability to otherwise refractory cellular environments. Collectively, PEnuc trades precision for higher activity and broader cellular range, representing a valuable option for editing in non-dividing cells, repair-restrictive contexts, or zygote models, where nickase-based approaches underperform.

### Dual-pegRNA systems

**Figure 3:**
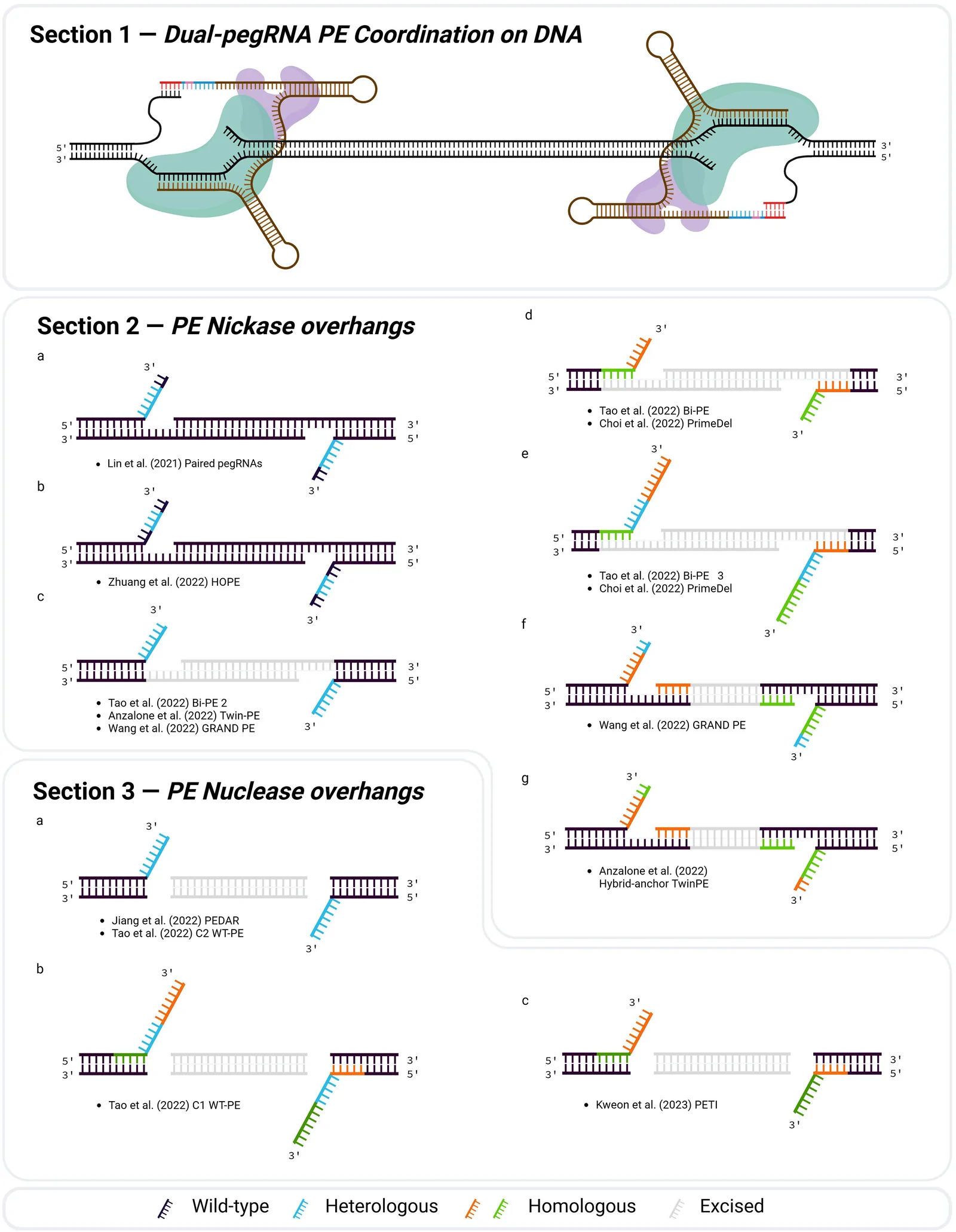
Dual-pegRNA systems and their repair strategies (1) PAM-facing dual-pegRNA PE with complementary overhangs. (2) This has been explored through numerous systems that differ in coordination of overhangs, including paired pegRNAs; HOPE; Bi-PE 1, 2, and 3; PrimeDel; GRAND-PE; and twinPE.^49^^,^^50^^,^^51^^,^^52^^,^^53^^,^^54^ (3) PE nuclease systems have also been explored, including PEDAR, WT-PE, and PETI.^55^^,^^56^^,^^57^

To expand the editing capacity of PE, dual-pegRNA systems employ two pegRNAs that simultaneously encode forward- and reverse-complementary repair templates on opposite DNA strands (Figure 3). Mechanistically, this arrangement enables the RTTs to generate complementary sequences that “bridge” across intervening genomic DNA, thereby bypassing one of the key limitations of single-pegRNA systems: competition between the 3′ edited flap and the unedited 5′ genomic strand. Instead, the *trans*-complementary RTTs anneal to one another, stabilizing the edited region and promoting integration. This cooperative mechanism allows correction not only of small mutations but also of larger insertions, deletions, and replacements that are otherwise inefficient. Importantly, because the final product is supported by complementary edits on both strands, dual-pegRNA strategies also evade MMR, which preferentially targets single-strand mismatches.

This concept was first validated in rice, where partially homologous dual-pegRNA systems significantly outperformed PE3 in both efficiency and precision for small insertions and point mutations.^49^ Following this proof of principle, dual-pegRNA platforms progressed into mammalian cells with the HOPE (homologous 3′ extension mediated prime editor) dual-pegRNA system, which tested both partially and fully homologous RTTs. In HEK293T, colorectal carcinoma, and other cell types, HOPE achieved higher efficiency and precision than single-pegRNA systems for short substitutions and insertions, including integration of a 24 bp epitope tag.^50^ Since then, dual-pegRNA designs have been adapted into several specialized platforms that enabled larger sequence modifications, such as exon-size deletions. This included the PRIME-Del platform, which enabled precise deletions up to 10 kb, with a 2-fold improvement in junction precision compared to dual-sgRNA Cas9 methods, and the Bi-PE (bi-direction prime editing) system, which coupled deletions with short insertions of up to 100 bp to further enhance deletion efficiency and junction precision.^51^^,^^52^ Alternatively, the GRAND (genome editing by RTTs partially aligned to each other but nonhomologous to target sequences within duo pegRNA) PE platform aligned RTTs to mediate insertions over 250 bp, though efficiency declined with increasing template size.^53^ Across these studies, HA design emerged as a critical determinant: externally flanking HA complementarity improved deletion precision, whereas overlap with intervening gDNA competed with the RTT and reduced efficiency.^52^^,^^53^ This principle was reinforced by direct comparison of the PRIME-Del and Anchor twinPE platforms, which showed that targeting flanking gDNA was roughly twice as efficient as targeting intervening sequence when creating intervening deletions.^54^ Further optimization has been achieved by stabilizing dual pegRNAs with epegRNA motifs, increasing correct editing efficiency by up to 50%.^54^ Beyond deletions, twinPE platforms have leveraged exogenous double-stranded DNA (dsDNA) donors to enable targeted large insertions. In this prime assembly (PA) approach, twinPE is designed to generate overhang sequences complementary to the flanking arms of the dsDNA donor, allowing the precise integration of intervening sequences via a Gibson-like assembly. This platform circumvents the current RTT length limitations to achieve insertions up to 11 kb.^58^

Dual-pegRNA strategies have also been integrated with PEnuc, which combines high DSB activity with the precision of complementary RTTs. By generating paired DSBs, these systems increase the likelihood of excising intervening sequences, thereby further reducing WT flap competition and enabling larger targeted rearrangements (Figure 3). One such system, PEDAR (PE-Cas9-based deletion and repair), achieved precise 8–10 kb deletions in mammalian cells where dual-Cas9 systems failed.^55^ The WT-PE dual-pegRNA approach extended this capacity to megabase-scale deletions and interchromosomal translocations with efficiencies of 3%–4%.^56^ Most recently, another PEnuc dual-pegRNA approach called PETI (prime editor nuclease-mediated translocation and inversion) demonstrated precise inversions and translocations at rates of up to 10% without scar formation, highlighting the potential of nuclease-based dual-pegRNA strategies for modeling structural variants.^57^

Collectively, dual-pegRNA approaches extend PE from short nucleotide modifications to 100 bp insertions and up to megabase-scale deletions and rearrangements, though efficiency and fidelity remain highly dependent on system design and target loci.

### PEI

Efficient insertions beyond 100 bp remain one of the most challenging applications for PE due to pegRNA size limitations. Prime editor integrase (PEI) systems address this bottleneck by coupling single- or dual-pegRNA PE with the site-specific recombination activity of Bxb1 integrase. Bxb1 is a serine recombinase derived from mycobacteriophage that catalyzes unidirectional recombination between two short recognition sites, 38 bp attB and 48 bp attP, to mediate precise integration of DNA cargo.^59^ In this framework, PE first installs a 30–50 bp recognition site (typically attB) at the genomic locus, while the donor plasmid carries the complementary attP site. Bxb1 integrase then recombines these sites, producing attL and attR junctions and stably integrating the donor cargo. This design bypasses RTT size restrictions and enables kilobase-scale insertions.^54^^,^^60^

Two PEI systems exemplify this approach. PASSIGE (prime-editing-assisted site-specific integrase gene editing) uses twinPE to install attB sites, followed by delivery of Bxb1 and donor vectors carrying attP, achieving up to 80% attB installation and ∼12%–17% insertion of a 5.6 kb donor in HEK293T cells.^54^ Alternatively, PASTE (programmable addition via site-specific targeting element) uses a Cas9-RT-Bxb1 fusion and the PE3 strategy to integrate attB and donor insertion in a single step, achieving 35%–45% precision with a 1 kb donor and 10%–20% with a 36 kb donor in primary hepatocytes.^60^ Although Bxb1 provides exceptional specificity, its limited efficiency constrains clinical translation. Directed evolution of Bxb1 via PACE yielded eeBxb1 (evovled and engineered Bxb1), enabling eePASSIGE to achieve a 4.2-fold improvement in integration efficiency over WT PASSIGE and outperform PASTE by an average of 16-fold across 12 genomic loci; however, integration at the therapeutically relevant COL7A1 locus—a target for dystrophic epidermolysis bullosa—remained modest in primary cells, underscoring that efficiency gains in transformed cell lines do not yet translate reliably to clinically relevant settings.^61^^,^^62^^,^^63^

Beyond Bxb1, PE has been paired with alternative recombinase chemistries tailored to organism and scale. PrimeRoot adapts the PEI logic to plants by combining an enhanced plant PE with high-activity Cre and FLP recombinases to enable targeted, DSB-independent large insertions. Third-generation PrimeRoot supports targeted 4.9 kb insertions up to 6.3% at a predicted safe-harbor locus in rice. As with att-site platforms, this strategy typically retains recombination-junction sequences at the integration boundaries.^64^

Complementary innovations have applied PE to Cre recombinase platforms in both plant and human cells, creating the programmable chromosome engineering (PCE) system. This PE recombinase system integrates asymmetric Lox sites to prevent recombination reversal, re-priming pegRNAs (Re-pegRNA) to erase residual scars, and AI-guided protein engineering to improve Cre activity. Distinct from previous PEI systems, PCE achieves scarless edits at scales from kilobases to megabases, enabling precise inversions, translocations, and replacements across plant and mammalian genomes, albeit at modest efficiency.^65^

Finally, bridge recombinases define a mechanistically distinct class of large-editing enzymes that do not require PE to pre-install att/lox landing sites.^66^ These RNA-guided recombinases use a bridge RNA (bRNA) to specify both genomic target and donor substrates, enabling programmable insertion, inversion, and excision. In engineered human-cell implementations (ISCro4), bridge recombination achieved up to 20% insertion with genome-wide specificity as high as 82% and supported the mobilization of genomic DNA at the megabase scale (reported inversions up to ∼0.93 Mb and excisions up to ∼0.13 Mb), including proof-of-concept excision of disease-relevant regulatory or repeat elements.

Together, PEI demonstrates how combining integrase systems with PE unlocks genome engineering at scales far beyond single-gene edits. While the requirement for multiple large components remains a challenge for delivery, these approaches highlight the adaptability of PE and its potential to bridge precision editing with large-scale genomic engineering. Emerging recombinase hybrids independent of PE further present attractive opportunities for large-scale and programmable modifications independent of recognition-site insertions.

As summarized in Table 2, recent architectural innovations in PE highlight the breadth of strategies available to expand its editing capacity. Collectively, PEnuc, dual-pegRNA systems, and PEI platforms extend the technology far beyond its original scope. These approaches move PE from a tool primarily suited for small, precise edits to one capable of supporting large insertions, deletions, and programmable rearrangements across diverse cellular contexts. Each carries distinct trade-offs between efficiency, precision, and delivery complexity, but together they demonstrate the versatility of PE when its architecture is reimagined rather than incrementally tuned.

**Table 2:** Prime editing systems and strategies overview

System	Key modification	Impact on editing/delivery	Reference
**Classic prime editors**			

PE1	fusion of wild-type M-MLV RT to Cas9 nickase (H840A).	baseline editor with low efficiency (∼1%–10%) due to suboptimal RT activity and limited edited-strand synthesis	Anzalone et al.^8^
PE2	engineered RT with five mutations (D200N, L603W, T330P, T306K, and W313F)	improves editing efficiency up to 5.1× over PE1 by stabilizing RT and enhancing successful edit incorporation	Anzalone et al.^8^
PE3	second sgRNA for a complementary strand nick 40–90 bp away	increases editing efficiency (∼4× over PE2) but introduces more unintended indels due to extra DNA cleavage	Anzalone et al.^8^
PE3b	variation of PE3 with the second nicking sgRNA directed only to the edited strand	decreases indels (∼13× lower than PE3) while preserving high editing rates	Anzalone et al.^8^
PE4 and PE5	addition of MLH1dn to PE2/PE3 to inhibit mismatch repair (MMR)	raises editing efficiency by up to 7.7× (vs. PE2) and 2× (vs. PE3) with fewer indels in MMR-proficient cells	Ferreira da Silva et al.^9^
PEmax	codon-optimized PE with extra NLS, R221K, and N394K mutations for improved nickase activity and structural changes	enhances nuclear localization and stability, boosting efficiency by 2.8× in HeLa cells	Ferreira da Silva et al. et al.^9^

**PE6 variants**			

PE6a	evolved Ec48 RT with higher activity and a smaller footprint (∼1.2 kb).	achieves 22× greater efficiency than wild-type Ec48, 3.7× over Marathon RT and is ∼810 bp smaller than PEmax	Doman et al.^18^
PE6b	evolved Tf1 RT specialized for substitutions	matches PEmax efficiency with a ∼1.5 kb size, enabling easier delivery	Doman et al.^18^
PE6c	evolved Tf1 RT plus processivity-enhancing mutations.	excels at complex edits, surpassing PEmaxDRNaseH for insertions	Doman et al.^18^
PE6d	truncated M-MLV RT with polymerase-enhancing mutations	improves performance by 2.3× over PEmaxDRNaseH for structured RTTs	Doman et al.^18^
PE6e	Cas9 domain mutations to strengthen DNA binding	attains 1.8× higher efficiency at certain loci	Doman et al.^18^
PE6f and PE6g	optimized Cas9 variants for better R-loop stabilization	improves efficiency in twinPE and large insertions	Doman et al.^18^

**Specialized prime editors**			

PE7	fuses La (1–194) RNA-binding domain to PEmax for pegRNA stabilization	delivers up to 21× higher efficiency vs. PEmax in U2OS cells, 5.5× better with epegRNAs, and strong performance in T cells/HSPCs	Yan et al.^14^
CMP-PE	incorporates chromatin-modulating peptides (CMPs) plus a dead sgRNA (dsgRNA)	increases editing ∼2× in heterochromatin and ∼5× in Igf2 and achieves 47% germline transmission in mouse embryos	Park et al.^67^
PASTE	PE3 fused to Bxb1 with attachment site pegRNAs (atgRNA)	surpasses HDR/HITI (∼30% with PASTE v.2), supporting up to 36 kb insertions; atgRNA v.2 enhances site stability	Yarnall et al.^60^
PASSIGE	PEmax, twin-pegRNA, and separate evolved Bxb1 recombinases (evoBxb1 and eeBxb1)	achieves 60% donor integration (3.2× improvement) and 9.1×–16× higher efficiency vs. PASTE	Pandey et al.^63^
PCE	PE Cre-Lox system with AI engineered recombinase and re-PE gRNAs to remove Lox scarring	enables kb-size insertions, deletions, and replacements in plants, achieving 20% efficiency of 300 kb inversion, 2% efficiency of 12 Mb inversion	Sun et al.^65^
PrimeRoot	engineered and plant optimized dualPE nickase epegRNA, paired with Cre or FLP recombinases for donor insertion; Sp, SpG, and SPrY Cas variants	up to 50% recombinase site insertion, achieving up to 11 kb insertions in rice	Sun et al.^64^
PE_Y18	yeast-based OrthoRep evolution adding A259D (nCas9) and K445T (RT)	improves efficiency by 3.5× over PEmax and 2.3× in HEK293T, with strong *in vivo* performance (mouse brain and liver)	Weber et al.^32^
PE2∗	PE2 optimized for codon sequence, NLS, and M-MLV RT architecture	yields 1.6× better efficiency vs. PE2 and 2.3× stronger RTT extension, improving precision across multiple cell types	Liu et al.^31^
miniPE	uses a compact CjCas9 (H559A) plus a truncated MMLV RT (removing 21 N-terminal and 200 C-terminal amino acids) to reduce total size to ∼4.5 kb	enables AAV packaging for *in vivo* delivery; exhibits modest editing (∼1%–1.5% *in vitro*) with improved efficiency under miniPE3, offering therapeutic potential	Lan et al.^68^
iPE	codon-optimized PE with superNLS and ΔRNase H	yields ∼1.3× better efficiency than PEmax, with iPE-N outdoing iPE-C in a PE3 setup using epegRNAs	Truong et al.^28^
Exo-PE	recruits a 5′-3′ exonuclease (T5) via a PP7 aptamer in the pegRNA	enhances editing efficacy, especially for insertions ≥30 bp, by removing 5′ flaps and increasing overall precision	Truong et al.^28^

**Nuclease prime editors**			

PEnuc	single-plasmid system containing all PE components plus a selection marker (also includes a nuclease-based variant, PEA1-Nuc)	reaches up to 95% editing in HEK293T, improving performance in K562, HeLa, and mouse embryos	Adikusuma et al.^44^
PRINS	SpCas9 nuclease-based prime editor leveraging homology-dependent and -independent DSB repair	boosts editing where conventional pegRNAs fail and prevents large deletions	Peterka et al.^45^
uPEn	combines a prime editor nuclease (PEn) with a 53BP1-inhibitory ubiquitin variant (UbvG08 (I44A)) via a P2A linker	improves precise editing efficiency and accuracy (up to 2.7×) with ∼90% product purity by mitigating error-prone NHEJ repair	Li et al.^46^

**Dual-pegRNA-based methods**			

HOPE	uses paired sense/antisense pegRNAs with inward-facing PAMs	increases efficiency and product purity while lowering indels compared to standard PE3	Zhuang et al.^50^
Bi-PE	converts both guide RNAs into pegRNAs for bidirectional priming	elevates editing up to 16× and precision by 60×, enabling large fragment edits	Tao et al.^52^
twinPE	employs twin pegRNAs that generate complementary 3′ flaps on opposite strands	supports large deletions, replacements, integrations, and inversions without DSBs	Anzalone et al.^54^
GRAND PE	uses pegRNA pairs with partially complementary RT templates that do not match the target	permits donor-free insertion up to ∼1 kb at high efficiency with minimal byproducts	Wang et al.^53^
PRIME-Del	two pegRNAs define nick sites and encode a repair junction for precise deletions	allows 5–10 kb deletions with few indels, plus short insertions and multiplex designs	Choi et al.^51^
PEDAR	active Cas9 nuclease fused to RT, with paired pegRNAs (pegF and pegR)	enables precise large-fragment deletion/insertion (up to 10 kb) without standard DSB repair	Jiang et al.^55^
WT-PE	PE2 nuclease plus paired pegRNAs with complementary sequences for bidirectional editing	facilitates large-scale edits (up to 16.8 Mb deletions) and interchromosomal translocations, enabling robust disease modeling of structural aberrations	Tao et al.^56^
PETI	PE2 nuclease plus paired pegRNAs for chromosomal rearrangements	allows modeling of complex translocations/inversions (e.g., NPM1-ALK) with fewer indels than Cas9	Kweon et al.^57^
Prime assembly	PE6c twin-epegRNA plus one or more linear donors for large insertions	capacity to perform Gibson-like insertions up to 11 kb in size	Liu et al.^58^

### Delivery of PE systems

Efficient delivery remains a central challenge for gene editing systems and is critical for accurate disease modeling, mechanistic studies, and therapeutic applications. An ideal delivery method must balance efficiency, tissue specificity, and safety while avoiding risks such as genomic integration, prolonged persistence, or immune responses.^69^ In the case of PE, this challenge is amplified by the system’s large protein size and multi-component architecture, which make cellular uptake and nuclear import particularly difficult. To overcome this, many delivery approaches have been explored, ranging from physical methods such as microinjection, hydrodynamic injection, and electroporation to viral and nanoparticle-based platforms that are advancing toward therapeutic application.

### *In vitro* delivery approaches

The complexity and comparatively large size of PE constructs exacerbate delivery challenges relative to conventional CRISPR systems. Designs such as PE3 or dual pegRNA require multiple components, increasing overall cargo size and reducing cellular delivery efficiency. *In vitro*, these systems can be streamlined into single DNA plasmids for efficient delivery by electroporation or lipofection in cultured cells and organoids, enabling robust mechanistic studies and accurate disease modeling.^44^^,^^63^ However, when considering translational applications, these strategies are inefficient. Lentiviral DNA delivery, while efficient for large cargos, carries the inherent risk of genomic integration; thus, safer alternatives are required when moving toward clinically relevant settings. Integrase-deficient lentiviral vectors (IDLVs) represent one such option, providing efficient delivery of large constructs, such as the 5.6 kb PASSIGE donor, while markedly reducing integration and toxicity in primary human fibroblasts.^63^ Although IDLVs have not yet been applied to PE delivery, their ∼10 kb packaging capacity positions them as a promising platform for future translation.

Beyond DNA-based formats, mRNA and ribonucleoprotein (RNP) delivery strategies have also been explored, offering the advantage of transient expression that reduces immune activation and limits off-target editing.^70^ Lipid nanoparticles (LNPs) are particularly attractive for mRNA delivery, given their generous size capacity and clinical precedent. Using LNPs, PE3 mRNA and pegRNA achieved up to 50% precise editing in a HEK293 reporter line, while in mouse Hepa1-6 cells, co-delivery of PE3 and chemically modified pegRNAs produced >60% insertion of a 4 bp sequence at the *Pcsk9* locus.^71^^,^^72^ In contrast, direct RNP delivery by nucleofection has generally produced lower efficiencies, most likely reflecting the short-lived stability of protein complexes. In human embryonic stem cells, correct editing remained below 5% across multiple loci, while in HEK293T cells, split-PE RNPs reached 15% precise correction of a 3 bp substitution at the VEGFA locus—still 4-fold lower than the corresponding mRNA delivery.^73^^,^^74^ To improve RNP editing efficiency, engineered virus-like particles (eVLPs) have been optimized for delivery, achieving up to 60% correction of a 4 bp substitution at the *Dnmt1* locus in Neuro-2A cells.^75^ Similarly, lentivirus-derived nanoparticles have been used to package and deliver PEmax RNPs into HEK293T cells, achieving ∼5% precise editing across multiple loci while maintaining a 4 day RNP turnover, thereby reducing off-target risk.^69^

*Ex vivo* delivery has already demonstrated therapeutic promise: in a tyrosinemia type I model, hepatic progenitors were corrected by PE3b DNA electroporation *ex vivo* (2.3% correction) before transplantation and repopulated the liver with 34.3% corrected alleles after 140 days.^76^ Similarly, *ex vivo* correction of patient-derived hematopoietic stem and progenitor cells (HSPCs) has demonstrated strong therapeutic potential: delivery of PE3max mRNA with epegRNAs targeting the HBB c.20 A>T mutation achieved ∼40% allele correction with preserved stem cell function, underscoring why hematopoietic stem cells remain the most advanced and tractable target for *ex vivo* gene therapies.^77^ By circumventing *in vivo* delivery barriers while maintaining a strong safety profile, *ex vivo* PE highlights a viable path for clinical translation, particularly in cell types amenable to isolation and transplantation.

### *In vivo* viral delivery approaches

Among viral approaches, AAV remains the most widely used due to its low immunogenicity, cell-type-specific serotypes, and clinical track record. However, AAV’s ∼4.8 kb packaging limit is insufficient for full-length PE, which requires ∼6.3 kb plus pegRNA/sgRNA cassettes. To overcome this, split-intein strategies have been developed: Cas9 is divided at defined amino acid sites and reconstituted in cells via inteins, with the best performance reported for Rma intein cleavage before amino acid position 1105 in a high-throughput screen.^78^ Reducing PE size has also been pursued through RT truncations that remove the RNaseH domain, shortening the construct by ∼600 bp without loss of activity.^33^^,^^34^^,^^35^^,^^36^ These innovations have enabled proof-of-principle *in vivo* applications, including correction of familial hypercholesterolemia (1.5% in mouse liver), Leber congenital amaurosis (6% in mouse retina), and tyrosinemia type I (1%–11% in mouse liver) via AAV8 delivery.^31^^,^^35^^,^^78^^,^^79^^,^^80^^,^^81^ Yet dual-vector strategies are still required, and efficiencies remain modest. More advanced designs, such as truncated PE-AAV (v3em PE-AAV) or directed-evolved PE6b delivered for twinPE, have demonstrated higher performance, achieving up to 40% cortical editing in the neonatal mouse brain.^18^^,^^82^ Other innovations include untethered nCas9/RT units, which bypass reconstitution, and coiled-coil heterodimerization of Cas and RT across dual-AAV vectors, both showing comparable efficiency to tethered systems.^74^^,^^83^

Adenovirus (AdV) offers a contrasting platform with much greater cargo capacity, sufficient for full-length PE. Despite its higher immunogenic profile and risk of integration, AdV has delivered impressive results: PE5max corrected 43% of sickle hemoglobin alleles in a murine sickle cell disease (SCD) model with phenotypic rescue and PE2 editing at the *Dnmt1* locus achieved 58% correction after tail-vein injection, far surpassing split-AAV (15%).^36^^,^^84^ These findings underscore both the potential and trade-offs of high-capacity viral systems.

### *In vivo* non-viral delivery approaches

Non-viral approaches provide additional flexibility for PE delivery across diverse contexts and avoid the risk of genomic integration. In mouse zygotes, cytoplasmic delivery of PE mRNA achieved robust editing under optimal conditions, supported by the high payload capacity of mRNA and the tolerance of early embryonic stem cells.^85^ Consistent with this, our own work demonstrated efficient editing with PEnuc mRNA delivered via cytoplasmic injection into zygotes.^44^ LNPs have also been used to deliver PE mRNA *in vivo*; retro-orbital intravenous injection of LNPs carrying PE3 and chemically modified pegRNAs yielded up to 8% editing of *Pcsk9* in liver hepatocytes following three weekly doses.^72^ However, daily dosing resulted in liver toxicity, underscoring the drawback of LNPs’ strong tropism for hepatocytes. For RNP delivery *in vivo*, microinjection of PE2 into zebrafish embryos yielded high efficiencies of up to 30% correct editing.^86^ eVLPs offer another non-viral strategy; however, they show marginal editing rates. Intracerebroventricular delivery of PE3 eVLPs produced a 2% correction of a 4 bp *Dnmt1* substitution in murine cortex, while subretinal injection of PE3b eVLPs achieved an ∼15% therapeutic correction of a 4 bp AAGT insertion in *Mfrp* of *rd6* mice.^75^

Overall, these strategies illustrate the breadth of approaches under development to deliver PE. Each platform balances efficiency, safety, and scalability in different ways, but all must contend with the fundamental challenge of PE’s size and complexity. Continued innovation in viral engineering, nanoparticle formulation, and *ex vivo* methodologies will be essential to achieving therapeutically relevant editing levels *in vivo*.

## Therapeutic applications of PE

Traditional CRISPR-based therapies are often restricted to generating small indels to disrupt coding sequences. This strategy has proven successful in certain contexts, such as *Casgevy*, the recently approved therapy for SCD that relies on Cas9-mediated disruption of the *BCL11A* enhancer to reactivate fetal hemoglobin (*HbF*).^84^ However, while indel-based editing can be highly effective for diseases where gene disruption is therapeutic, for many genetic disorders, simply inactivating a gene provides little or no therapeutic benefit. Instead, precise recoding of pathogenic alleles back to functional sequences is required—an outcome that conventional CRISPR systems struggle to achieve efficiently.

PE, with its ability to directly rewrite pathogenic alleles into non-pathogenic sequences, offers a more versatile and precise therapeutic strategy for the >75,000 disease-associated human variants spanning hundreds of disorders and mutation classes.^87^ Beyond restoring the WT sequence, PE also enables strategies for codon reframing and precise, large sequence rearrangements, which expand the therapeutic potential of genome editing. Below, we discuss representative PE applications across major target tissues, selecting one well-studied disease context per tissue, with additional applications summarized in Table 3.

**Table 3:** Disease applications of prime editing systems

Edit type	Disease/disorder (mutation; gene target)	PE system	Efficiency (cell type and outcome)	Delivery, reagent, and selection	Reference
***In vitro***					

Base substitution, deletion	sickle cell disease (HBB c.20 A>T, p.E6V), Tay-Sachs (HEXA 1278+TATC), PRNP (G127V)	PE3	∼60% HBB correction, 33% HEXA, and 53% PRNP in HEK293T cells	lipofection of plasmid DNA; no selection	Anzalone et al.^8^
Base substitution	sickle cell disease (HBB c.20 A>T, p.E6V)	PE3max + epegRNA	15%–41% correction in patient-derived HSPCs, maintaining 42% correction in downstream human erythroblasts and reticulocytes following murine transplantation	electroporation of mRNA (PE) + synthetic epegRNA + sgRNA; no selection	Everette et al.^77^
Base substitution	cystic fibrosis (CFTR ΔF508)	PE3/PE5/PE6c/PEmax + epegRNA	up to 58% correction in immortalized bronchial epithelial cells; ∼25% correction in patient-derived airway epithelial cells	electroporation of mRNA (PE) + synthetic epegRNA + ngRNA; no selection	Sousa et al.^88^
Base substitution/insertion	cancer modeling (TP53 R249S) and cystic fibrosis (CFTR ΔF508, R785∗)	PE3	∼25% efficiency in colon organoids; up to 97% in hepatocyte organoids (via clonal expansion); editing was less successful in CFTR organoids	electroporation of plasmid DNA; hygromycin; functional selection (Nutlin-3 for TP53; forskolin swelling for CFTR)	Geurts et al.^89^
Base substitution	dilated cardiomyopathy (RBM20 c.1906 C>A, p.R636S)	PE3bmax + epegRNA	up to ∼40% correction in iPSCs	nucleofection of plasmid DNA; FACS (GFP^+^/mCherry^+^)	Nishiyama et al.^90^
Deletion	Duchenne muscular dystrophy (WT)	twinPE	up to 40% efficiency of a DMD exon 51 589 bp deletion plus 38 bp Bxb1 attB insertion in HEK293T cells (twinPE yields improved reframing with fewer indels compared to Cas9)	lipofection of plasmid DNA; no selection	Anzalone et al.^54^
Large deletion	Duchenne muscular dystrophy (WT)	WT-PE	4%–7% Ex17–55 in-frame deletion in HEK293T cells, proposed as a universal treatment	lipofection of plasmid DNA; no selection	Tao et al.^56^
Base substitution	Duchenne muscular dystrophy (DMD exon 59 c.8713 C>T)	PE2/PE3	∼22% modification in patient-derived myoblasts, with dystrophin restoration in differentiated myotubes	electroporation of plasmid DNA; no selection	Happi Mbakam et al.^91^
Insertion	Duchenne muscular dystrophy (DMD ΔEx51)	PE3	∼20%–54% precise +2 nt insertion in iPSC-derived cardiomyocytes; restoration of dystrophin with improved contractile function; correction levels of 20%–40%	nucleofection of plasmid DNA; no selection	Chemello et al.^92^

***In vivo***					

Base substitution	alpha1 antitrypsin deficiency (SERPINA1 c.1024 G>A, p.E342K)	split-intein PE2∗ (PE3)	∼0.6% correction at 2 weeks, rising to ∼3.1% in adult mouse liver; clonal expansion observed in hepatocytes	dual-AAV8 (hydrodynamic tail-vein injection)	Liu et al.^31^
Base substitution	liver cancer modeling (CTNNB1 S45F)	PE2∗ + nicking sgRNA	>80% of tumor DNA harbored the S45F edit in adult mouse liver; multiple hepatic tumors observed	hydrodynamic tail-vein injection of plasmid DNA	Liu et al.^31^
Large deletion	phenylketonuria (PAH c.835 T>C, p.F263S)	PE2 with ΔRNaseH/PE3b	average 11% correction in neonatal mouse liver, up to 17% in some mice; normalization of blood phenylalanine levels	adenoviral vector/AAV8	Böck et al.^36^
Base substitution	phenylketonuria (PAH c.1222 C>T, p. Arg408Trp)	PE3bmax	up to 52% targeted integration in mouse liver; normalization of blood phenylalanine levels	dual-AAV8 (intravenous [i.v.] injection)	Brooks et al.^93^
Base substitution	sickle cell disease (HBB c.20 A>T, p.E6V)	PE5max	∼43% correction of HbS and phenotypic rescue in mice	adenoviral vector (HDAd, i.v. injection)	Li et al.^84^
Base substitution	retinitis pigmentosa (PDE6B c.1678 C>T, p.R560C)	PE-SpRY	∼70% precise editing in retinal cells; substantial restoration of visual function in treated mice	dual-AAV subretinal injection	Qin et al.^80^
Base substitution	retinitis pigmentosa (PDE6B c.1041 C > A, p.Y347×)	PE3 + epegRNA	∼26% correction in total eye lysate with significant functional rescue	dual-AAV2 subretinal injection	Fu et al.^94^
Insertion	autosomal recessive retinal degeneration (Mfrp 4 bp deletion rd6)	PE3b + epegRNA	∼15% correction in retinal cells	PE-eVLP subretinal injection of mRNA (PE) + synthetic RNA	An et al.^75^
Base substitution	Leber congenital amaurosis (Rpe65 c.130 C>T)	PE3b + epegRNA	∼7% precise editing in retinal cells; partial restoration of visual function in mouse models	PE-eVLP subretinal injection of mRNA (PE) + synthetic RNA	An et al.^75^
Base substitution	Leber congenital amaurosis (Rpe65 c.130 C>T)	PE2	∼7.7% correction in mouse RPE and phenotypic rescue	subretinal injection (AAV2)	Jang et al.^81^
Base substitution	Leber congenital amaurosis (Rpe65 c.130 C>T)	split-intein PE3	∼11% correction in mouse RPE and phenotypic rescue	subretinal injection (dual-AAV8)	She et al.^78^
Base substitution	hereditary tyrosinemia type I (FAH c.706 G>A)	PE2 and PE3	∼11.5% editing in mouse liver; expansion of FAH+ hepatocytes up to ∼61% by immunohistochemistry (IHC), with restored liver function	hydrodynamic injection of plasmid DNA	Jang et al.^81^
Base substitution	hereditary tyrosinemia type I (FAH c.706 G>A)	split prime editor (sPE, PE3)	∼1.3% editing in adult mouse liver; sufficient for phenotypic rescue	dual-AAV (tail-vein injection)	Liu et al.^74^
Large deletion	hereditary tyrosinemia type I (FAH ΔExon5, 1.38kb insert)	PEDAR	initial ∼0.76% FAH+ cells repopulated mouse liver, resulting in ∼78% accurate deletion of the 1.3 kb mutation and phenotypic rescue	hydrodynamic injection of plasmid DNA	Jiang et al.^55^
Base substitution	hereditary tyrosinemia type I (FAH c.706 G>A)	PE3b	∼2.3% editing (∼13.1% in clones); *ex*-*vivo*-corrected CdHs repopulated the liver (∼34%) and improved survival in HT1 mice	*ex vivo* electroporation of plasmid DNA + intrasplenic transplant	Kim et al.^76^
Base substitution	recessive dystrophic epidermolysis bullosa (COL7A1 c.3631 C>T, p.Q1211×; c.2005 C>T, p.R669×)	PE3	∼5% and ∼10% correction of c.2005 C>T and c.3631 C>T in patient-derived fibroblasts, with functional restoration of type VII collagen expression; corrected fibroblasts transplanted into mice maintained this expression	electroporation of plasmid DNA + skin grafting	Hong et al.^95^

**Clinical application**					

Insertion	chronic granulomatous disease (NCF1 c.75 delGT)	PE3 with 2′-O-methyl pegRNA + gRNA	*ex vivo* correction was high, with 68% (participant 1) and 91% (participant 2) of colony-forming cells carrying at least one corrected allele and 13% and 23% GTGT reads in the infused bulk products, respectively; post-infusion, neutrophil function recovered rapidly, reaching 83% dihydrorhodamine-positive neutrophils at day 30 (participant 2) with sustained NADPH oxidase activity for 6 (participant 1) and 4 (participant 2) months	*ex vivo* electroporation of mRNA (PE) + chemically modified synthetic RNA (2′-O-methyl epegRNA + sgRNA) into autologous HSPCs; i.v. infusion via central venous catheter; no selection	Gori et al.^96^

### Duchenne muscular dystrophy

Duchenne muscular dystrophy (DMD) is a severe, progressive X-linked neuromuscular disorder that affects ∼1 in 5,000 live male births and is characterized by early loss of ambulation followed by premature mortality due to cardiac and respiratory failure.^97^ The disease is caused by mutations in the dystrophin gene (∼2.3 Mb), most commonly frameshift mutations that disrupt the open reading frame. Current treatment strategies—including exon-skipping antisense oligonucleotides, AAV-mediated gene supplementation, and corticosteroids—can delay disease progression but remain only partially effective and are not curative.^97^

A key feature of dystrophin biology is that much of the central rod domain is structurally redundant: while it contributes to protein length and flexibility, it can tolerate large internal truncations provided that the reading frame is preserved.^98^ This is exemplified in patients harboring in-frame deletions, who often retain partial dystrophin function and consequently present with the milder Becker muscular dystrophy phenotype.^99^ This property creates opportunities for genome editing approaches aimed at reframing dystrophin transcripts. Conventional CRISPR-Cas9 editing strategies have attempted to restore the open reading frame by generating localized indels.^98^^,^^100^^,^^101^ However, because indels occur stochastically, only a subset of outcomes are frame restorative, limiting predictability and therapeutic efficiency. Other approaches have explored exon skipping, in which Cas9-mediated excision of a suitably sized exon restores the transcript’s reading frame.^98^^,^^102^^,^^103^ However, this strategy requires coordinated activity at two gRNAs and yields variable efficiency across genomic contexts.

Alternatively, PE offers a more precise and programmable means to reframe DMD.^98^^,^^104^ For example, Chemello et al. used PE3 to introduce a therapeutically reframing 2 bp insertion in exon 52 of induced pluripotent stem cell (iPSC)-derived cardiomyocytes from a ΔEx51 mouse model—one of the most common DMD mutations, achieving 54% allele correction, 40% of normal *dystrophin* expression, and functional recovery.^92^ In patient-derived myoblasts, Happi et al. corrected 21% of c.8713 C>T mutation alleles with PE5, restoring up to 42% of dystrophin protein.^91^ However, beyond mutation-specific correction, Tao et al.^56^ demonstrated a “universal” DMD therapy approach using dual-pegRNA WT-PE to delete exons 17–55, which encompass most pathogenic variants. Although efficiency was modest (7% in HEK293T), this strategy preserved reading frame and functional domains while demonstrating the potential of unique PE-based therapeutic strategies.^56^^,^^105^ Comparable exon-removal strategies have previously been explored with intron targeting dual-gRNA Cas9 nucleases, including excision of the exon 45–55 mutational hotspot, which restored dystrophin expression *in vitro*.^106^^,^^107^ While pegRNA-mediated repair may offer greater precision at edit junctions than dual-gRNA nuclease approaches, such precision is largely redundant in intronic regions. Thus, the true therapeutic advantage of PE in this context lies in its capacity to achieve reliable reframing. Rigorous side-by-side comparisons of these platforms will be necessary to clarify relative efficiencies, precision, and translational potential.

Together, these studies demonstrate the promise of PE for predictable reframing compared to earlier CRISPR approaches. Yet translation remains challenging: most data are derived from proliferative cell models rather than post-mitotic muscle, and even successful reframing yields Becker muscular dystrophy rather than full restoration. Achieving therapeutic benefit will require overcoming efficiency and delivery barriers in skeletal and cardiac muscle, tissues less permissive to PE.^26^^,^^27^

### Hereditary tyrosinemia type I

Hereditary tyrosinemia type I (HT1) is a rare autosomal-recessive disorder caused by loss-of-function mutations in the *fumarylacetoacetate hydrolase* (*FAH*) gene. Deficiency of FAH results in the accumulation of toxic tyrosine metabolites, driving progressive hepatic and renal failure, recurrent neurological crises, and a markedly increased risk of hepatocellular carcinoma (HCC).^108^^,^^109^ HT1 has an occurrence of approximately 1 in 100,000 live births.^109^ Current standard-of-care therapy with nitisinone alleviates hepatotoxicity by inhibiting an upstream step in the tyrosine degradation pathway, thereby preventing accumulation of toxic intermediates. Although lifesaving, this therapy must be combined with strict dietary control, requires lifelong administration, and does not eliminate the risk of HCC.^109^

To improve therapeutic options for HT1, PE has been explored through a range of different approaches to treat the common *FAH* c.706 G>A point mutation mouse model. Jang et al. achieved 11.5% allele correction of the pathogenic point mutation in the liver via hydrodynamic injection of PE3 plasmids, translating into 61% FAH-positive hepatocytes due to selective expansion of corrected cells.^81^ Liu et al. used split-AAV to deliver PE3, correcting 1.3% of *FAH* c.706 G>A alleles but still achieving robust FAH protein restoration.^74^ *Ex vivo* approaches have also been tested for this model, where electroporated PE3b into hepatic progenitors achieved 2.3% correction, and after transplantation into mice, repopulation yielded 34.3% corrected alleles *in vivo*.^76^ Alternatively, Jiang et al. applied the dual-pegRNA PEDAR system to restore 1.4 kb in *FAH* ΔExon5 mice, rescuing FAH expression and hepatocyte patches *in vivo*.^55^

These results underscore a key principle: even modest editing efficiencies can produce therapeutic benefit in the liver through clonal expansion of corrected hepatocytes. However, PE must also address extrahepatic manifestations of HT1, including renal and neurological involvement, where selective repopulation of corrected cells does not occur. Improving systemic delivery and efficiency will therefore be critical for curative HT1 outcomes.

### SCD

SCD predominantly arises from a single *HBB* c.20 A>T missense mutation that distorts hemoglobin structure, causing red blood cell sickling, anemia, vaso-occlusive crises, and multi-organ complications. Each year, ∼275,000 infants are born with SCD worldwide.^110^^,^^111^ Current management—blood transfusions, hydroxyurea, and bone marrow transplantation—offers partial control, while CRISPR-based therapies targeting *BCL11A* to reactivate *HbF* represent a major breakthrough.^112^^,^^113^ However, these approaches act indirectly, rely on *ex vivo* HSPC manipulation, and do not correct the underlying *HBB* mutation.

Alternatively, PE has the potential to directly restore the causative mutation and reinstate adult hemoglobin. This has been explored *in vitro* and *in vivo*, targeting the *HBB* c.20 A>T point mutation across a range of PE platforms. In HEK293T cells, PE3 corrected up to 60% of HBB alleles.^8^ *Ex vivo* correction of patient-derived HSPCs with PE3max and epegRNAs achieved ∼40% allele correction, and transplantation into immunodeficient mice preserved high editing in erythroid lineages with phenotypic rescue.^77^ *In vivo*, adenoviral delivery of PE5max corrected 43% of HSC alleles in an SCD mouse model, with minimal indels, no off-target activity, and a rescued phenotype.^84^

These findings highlight PE’s potential for direct, durable correction of SCD at its root cause. Compared with indirect strategies, PE provides a path to curative therapy, though challenges in the safe, efficient delivery to HSPCs remain.

### Leber’s congenital amaurosis

Leber’s congenital amaurosis (LCA) comprises severe, early-onset retinal dystrophies that cause blindness in ∼1 in 30,000 individuals.^114^ LCA is typically diagnosed in infancy and is characterized by profound vision loss or blindness due to mutations in genes essential for photoreceptor development and function. Existing options are limited: the FDA-approved AAV gene therapy Luxturna restores *RPE65* function but only in a small subset of patients and does not halt degeneration.^114^ CRISPR clinical trials (EDIT-101) aimed at excising *CEP290* mutations, the primary contributor of LCA, using dual-cut strategies, have shown limited responses.^115^^,^^116^

In contrast, PE enables precise correction without DSBs. In a *Rpe65* c.130 C>T mouse model of LCA, AAV delivery of PE2 corrected 6.4% of alleles in retinal pigment epithelium (RPE) cells and restored retinal electrophysiology, despite a low transduction rate of 23%.^81^ eVLP delivery of PE3b to correct this point mutation achieved comparable efficiencies.^75^ Although overall editing rates remain low, estimates suggest that correction of ∼10% of cone photoreceptors could restore near-normal vision, underscoring the clinical potential of even modest PE activity in the retina.^117^

### Translation to the clinic

Recently, PE entered the clinic with PM359 for the treatment of chronic granulomatous disease, a rare inherited immunodeficiency affecting approximately 1 in 200,000 births, caused by pathogenic variants in genes encoding subunits of the phagocyte NADPH oxidase complex.^118^ Loss of NADPH oxidase activity impairs reactive oxygen species generation required for microbial killing, leading to recurrent life-threatening bacterial and fungal infections and inflammatory complications. PM359 is an autologous CD34+ hematopoietic stem and progenitor cell product in which the recurrent *NCF1* delGT pathogenic variant is corrected *ex vivo*. Manufacturing used electroporated PE3 mRNA with chemically modified pegRNA and gRNA and achieved substantial on-target correction, with 68% and 91% of colony-forming cells carrying at least one corrected allele and 13% and 23% GTGT reads in the infused bulk cell populations, followed by busulfan conditioning and rapid hematologic recovery after infusion.^96^ Functional rescue was robust, with 69% and 83% dihydrorhodamine-positive neutrophils at day 30 and sustained neutrophil oxidase activity through 6 and 4 months of follow-up, respectively, alongside no serious adverse events attributed to PM359. Preclinical profiling further supported a favorable product purity profile, with unintended edits in under 2% of colony-forming cells, no evidence of genome sequence loss or chromosomal abnormalities, and no detectable off-target editing, collectively underscoring the feasibility of clinical-scale PE for therapeutic development.

Across diverse disease contexts, PE offers distinct therapeutic advantages: transcript reframing in DMD, selective expansion in HT1, direct causative correction in SCD, and single-nucleotide precision in LCA. While delivery and efficiency remain limiting factors, these studies demonstrate that PE is uniquely positioned to expand therapeutic genome editing precision beyond the capabilities of current CRISPR approaches, as outlined in Table 3.

## Safety considerations of PE therapy

PE is often viewed as a safer alternative to nuclease-based CRISPR because it can introduce defined sequence changes without an intended DSB. However, therapeutic deployment still requires rigorous evaluation across four risk domains: off-target activity, on-target product purity, large-scale genome integrity, and immunogenicity of the foreign components.

PE generally exhibits lower off-target editing than sgRNA-guided nucleases, consistent with a higher mechanistic barrier to productive editing that requires coordinated nicking and reverse transcription with multiple layers of guide-target complementarity (spacer, PBS, and template).^8^ Nonetheless, off-target risk is not eliminated and must be empirically profiled in the relevant cell type and delivery format. Because traditional PE is nick dependent rather than DSB dependent, off-target profiling requires assays tailored to this mechanism. Established DSB-centric methods have clear limitations in this context: GUIDE-seq (genome-wide, unbiased identification of DSBs enabled by sequencing), which captures NHEJ-mediated integration of a double-stranded oligonucleotide at blunt-ended breaks, cannot label nicked loci, while CIRCLE-seq (circularization for *in vitro* reporting of cleavage effects by sequencing)—which selectively circularizes sheared genomic DNA for Cas9 nuclease cleavage and adapter ligation—is inherently dependent on DSB activity and is not directly applicable to nicking editors.^119^^,^^120^ To address this gap, PE-specific discovery approaches exploit the editor’s own RT to mark off-target loci: both PE-tag and TAPE-seq (tagmentation of prime editor sequencing) use a tagging pegRNA to direct RT-mediated insertion of an amplification tag at sites of PE activity genome wide, enabling deep-sequencing identification of off-target loci.^121^^,^^122^ A limitation of these RT-dependent approaches is that loci where nicking occurs without subsequent successful reverse transcription will be invisible to tag-based detection, potentially underrepresenting the true nicking frequency at off-target sites. PEG-seq (3′-end ligation sequencing) circumvents this by directly ligating sequencing adapters to the exposed 3′ hydroxyl at nicked loci in extracted genomic DNA, detecting nicking activity independently of RT incorporation.^123^ Together, these approaches offer complementary coverage of PE off-target activity, though direct benchmarking across matched cellular contexts will be needed to establish their relative sensitivity and inform consensus profiling standards.

Importantly, safety is also determined by which alleles are produced at the intended locus. Although PE is designed to template a specified edit and can do so with substantially higher product purity than HDR, the degree of purity is context dependent and varies across targets and models.^8^ Competing repair outcomes can yield on-target byproducts, including local indels, partial template incorporation, or complex alleles, which may reduce efficacy or introduce new pathogenic variants. A related translational concern is loss of re-targetability, where disruptive byproducts alter the protospacer or PAM and impede repeat dosing or iterative correction. Accordingly, therapeutic PE development requires close inspection of allelic outcome distributions, not only nominal edit rates.

Finally, avoidance of an intended DSB should reduce, but not abolish, risks of structural variation. Genome integrity profiling remains prudent for clinically relevant settings, particularly in primary cells, repeat-rich loci, or high-expression delivery regimes. In parallel, immunogenicity against editor proteins and delivery vehicles can aggravate treatment and complicate re-administration. This has motivated strategies that minimize immunogenic epitopes present in the editor and reduce exposure duration and dose while maintaining therapeutic editing levels.^124^

Overall, PE safety will be defined by context-specific control of off-target activity, on-target byproducts, structural variation, and immunogenicity. Accordingly, safety benchmarking in therapeutically relevant primary cells should be pursued in parallel with efficacy throughout therapeutic development.

## Conclusion and future prospects

PE has emerged as a transformative genome engineering platform, enabling precise and programmable sequence changes without the need for DSBs or donor templates. Rapid advances in RT engineering, pegRNA architecture, Cas variant development, and delivery modalities have expanded both editing performance and the addressable mutational landscape. Proof-of-concept studies across hepatic, hematopoietic, muscular, and retinal systems have underscored its capacity to correct pathogenic variants that remain difficult to treat with conventional nuclease-based approaches, and this promise has now begun to translate clinically with the first-in-human PE trial, which has thus far reported functional restoration without PE-attributed serious adverse events.

Despite this momentum, key barriers remain. Editing efficiency is still constrained in post-mitotic tissues, delivery is complicated by the size and stoichiometry of PE components, and outcomes can vary substantially with cell-type-specific repair context. Addressing these limitations will require continued innovation in compact and modular editor designs, recombinase-integrated architectures for larger-scale genome rewriting, and delivery vehicles optimized for tissue-specific access and transient expression. Parallel progress in safety benchmarking, including rigorous quantification of on-target byproducts, structural variation, and immunogenicity, will be essential as PE moves beyond *ex vivo* settings into systemic *in vivo* applications.

Together, ongoing mechanistic insight and engineering innovation are positioning PE to move therapeutic genome editing beyond gene disruption toward precise, durable correction of disease-causing alleles. The field’s shift from preclinical demonstration to early clinical validation marks an inflection point, and continued advances in efficiency, delivery, and safety will determine how broadly PE can redefine the next generation of genetic medicines.

## Acknowledgments

F.A. is supported by a Discovery Early Career Research Award from the 10.13039/501100000923Australian Research Council and the 10.13039/501100000925National Health and Medical Research Council Investigator Fellowship. We thank the PQT lab for the ongoing support and feedback on the manuscript.

## Author contributions

Conceptualization, C.L. and F.A.; data generation, C.L.; writing – original draft, C.L., P.T., and F.A.

## Declaration of interests

The authors have no competing interests to declare.
